# Practical Measures of Integrated Information for Time-Series Data

**DOI:** 10.1371/journal.pcbi.1001052

**Published:** 2011-01-20

**Authors:** Adam B. Barrett, Anil K. Seth

**Affiliations:** Sackler Centre for Consciousness Science and School of Informatics, University of Sussex, Brighton, United Kingdom; Indiana University, United States of America

## Abstract

A recent measure of ‘integrated information’, Φ_DM_, quantifies the extent to which a system generates more information than the sum of its parts as it transitions between states, possibly reflecting levels of consciousness generated by neural systems. However, Φ_DM_ is defined only for discrete Markov systems, which are unusual in biology; as a result, Φ_DM_ can rarely be measured in practice. Here, we describe two new measures, Φ_E_ and Φ_AR_, that overcome these limitations and are easy to apply to time-series data. We use simulations to demonstrate the in-practice applicability of our measures, and to explore their properties. Our results provide new opportunities for examining information integration in real and model systems and carry implications for relations between integrated information, consciousness, and other neurocognitive processes. However, our findings pose challenges for theories that ascribe physical meaning to the measured quantities.

## Author Summary

A key feature of the human brain is its ability to represent a vast amount of information, and to integrate this information in order to produce specific and selective behaviour, as well as a stream of unified conscious scenes. Attempts have been made to quantify so-called ‘integrated information’ by formalizing in mathematics the extent to which a system as a whole generates more information than the sum of its parts. However, so far, the resulting measures have turned out to be inapplicable to real neural systems. In this paper we introduce two new measures that can be applied to both realistic neural models and to time-series data garnered from a broad range of neuroimaging and electrophysiological methods. Our work provides new opportunities for examining the role of integrated information in cognition and consciousness, and indeed in the function of any complex biological system. However, our results also pose challenges for theories that ascribe a direct physical meaning to any version of integrated information so far described.

## Introduction

How can the complex dynamics exhibited by networks of interconnected elements best be measured? Answering this question promises to shed substantial new light on many complex systems, biological and non-biological. Neural systems in particular are characterized by richly interconnected elements exhibiting complex dynamics at multiple spatiotemporal scales [1], which have been associated with a variety of behavioral, cognitive, and phenomenal properties [2], [3], [4]. Characterizing dynamical complexity for such systems therefore presents a key challenge for developing new theoretical accounts [5] and for designing and evaluating new experiments. A common and attractive intuition is that dynamical complexity consists in the coexistence of *differentiation* (subsets of a system are dynamically distinct) and *integration* (the system as a whole exhibits coherence) in a system's dynamics. Applied to neural systems, this intuition may underpin notions of cognitive and behavioral flexibility. A system that is able to respond specifically and selectively to a broad range of stimuli, in an integrated way, may require conjoined functional integration and differentiation [6], [7]. More ambitiously, the intuition may also characterize basic aspects of conscious experience [8]. At the phenomenal level, each conscious scene is composed of many different parts and is different from every other conscious scene ever experienced (differentiation), yet each conscious scene is experienced as a coherent whole (integration). Therefore, dynamical complexity in neural systems may actually *account for* (and not merely correlate with) fundamental aspects of consciousness [9].

Several measures now exist which operationalize the above intuition under different assumptions and with varying practical applicability [5]. In this paper, we critically evaluate ‘integrated information’ (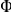) [10], [11], a candidate measure that has received significant recent attention, especially in the domain of consciousness science [12], [13], [14], [15]. We present new versions of this measure that are both theoretically well-grounded and, in contrast to previous versions, practically applicable given time-series data. 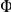 has been proposed as a measure of the amount of information that is integrated by a system, where ‘information’ reflects the differentiated states of a system and ‘integration’ their global cohesion. According to the ‘integrated information theory of consciousness’ (IITC), this quantity is identical to the quantity of consciousness generated by the system; in other words, on the IITC, consciousness *is* integrated information [12], [14]. This dramatic claim invites a close examination of the in-principle and in-practice properties of 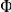.

A first version of 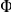 (which we call 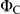, ‘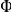-capacity’,) was conceived as a measure of the *capacity* of a system to integrate information, and did not take into account time or changing dynamics [10], [12]. Also, measuring 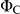 requires flexible, repeated, and reversible perturbation of arbitrary system subsets, which is infeasible for non-trivial systems (except in simulation). We do not discuss this measure any further. Recently, a new version of 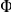 has been introduced in the context of the IITC, which we call 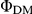, ‘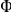-discrete/Markov’ [11]. In contrast to 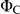, 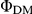 is defined for systems of discrete elements that evolve through time with Markovian transitions. Specifically, 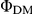 measures the information generated when a system transitions to one particular state out of a repertoire of possible states, but only to the extent that this information is generated by the whole system, over and above the information generated independently by the parts [11]. Importantly, 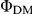 measures information as reduction in entropy from a prior *maximum entropy* distribution, which is taken to represent the repertoire of possible states.

It has been shown, using simulations, that 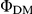 behaves consistently with several intuitions about dynamical complexity [11]. In particular, high values of 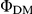 are generated by networks that exhibit both differentiation and integration in their dynamics. However, 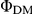 is defined only for idealized discrete Markovian systems (a Markovian system is one for which the future depends only on the present, and not on the past). This in-principle restriction severely limits its in-practice applicability because complex biological systems are typically continuous (or are measured as continuous) and are non-Markovian). This limitation in turn imposes a serious obstacle for developing and evaluating theories, such as the IITC, which depend on quantifying integrated information.

In this paper we introduce an alternative measure of integrated information, 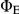 (‘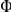-empirical’), which is applicable to time-series data, and to continuous or discrete stochastic systems, Markovian or otherwise (and without perturbation of the studied system). These key features arise because 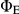 is based on the reduction in Shannon entropy from the empirical, as opposed to the maximum entropy, distribution. Our basic formulation of 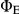 therefore addresses the in-principle restrictions of 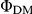 mentioned above. 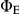 is best suited for application to stationary systems, for which it provides a single value for a given stationary epoch. However, its in-practice applicability still faces the difficulty of accurately estimating entropies from limited data. This is a problem that scales poorly as the number of elements (variables) increases, especially for continuous systems [16]. Confronting this problem, we show that when states are Gaussian distributed, 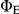 can be computed directly from empirical covariance matrices, rendering it extremely easy to apply in practice for these systems. Meanwhile, for non-Gaussian systems, we introduce a second measure, 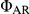 (‘auto-regressive 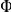
’), which is based on auto-regressive prediction error. 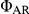 can be understood as measuring how well the present state of a system predicts some previous state, but only to the extent that predictions based on the whole outstrip predictions based on the parts considered independently. 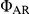 and 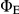 are constructed analogously, and indeed for Gaussian systems we are able to show, using a connection between linear regression and information theory [17], [18], that they are precisely equivalent. Recognizing this equivalence allows us to interpret 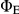 in the same way as 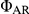, i.e., in terms of predictive ability. Importantly, although for non-Gaussian systems 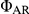 and 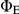 may differ, the former remains easy to measure in practice from empirical covariance matrices.

The difference between 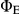/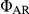 and 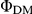 is not only a matter of practical applicability. Using the empirical distribution as opposed to the maximum entropy distribution substantially changes possible interpretations of the measure. According to 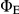, integrated information is a measure of a *process*, since the empirical distribution is a characterization of the actual behavior of the system. According to 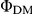 integrated information is to some extent a measure of *capacity* [14], since the maximum entropy distribution is maximally agnostic about the behavior of the system, representing instead its potential or capacity.

The above distinction carries implications for theories, such as the IITC, that ascribe physical meaning to measures of integrated information. Under the IITC, consciousness is explicitly characterized in terms of the capacity of a system [14], and not, following William James [19], as a process. Our new measures imply a Jamesian modification of the IITC by considering consciousness as a process; they also challenge the identity relation between consciousness and integrated information assumed in the IITC. More generally, many other brain-based phenomena are best considered in terms of process rather than capacity, and may admit useful interpretations in terms of integrated information. For example, multi-modal binding and perceptual categorization [20] could involve integrated information in the perceptual domain, and action selection (decision making) [21] may require the integration of sensory, cognitive and motor processes, while retaining differentiation among competing alternatives. In these and other cases, having a measure of integrated information framed in terms of process, that is practically applicable to time-series data, will permit the formulation of testable hypotheses and synthetic models relating information integration to cognitive and neural operations.

## Results

The ‘Results’ section is organized as follows. In the ‘Notation, conventions and preliminaries’ section we lay out our notation and introduce some necessary mathematical concepts. In the section ‘The previous measure, 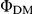
’ we review 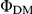 using our current notation, noting its limitations especially with respect to discrete Markovian systems. The section ‘The new measure, 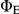
’ describes the new measure 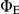 and provides practical recipes for its computation either numerically from time-series or analytically, given a generative model of the system, both under Gaussian assumptions. We note that for non-Gaussian systems 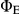 remains well-defined even if it is more challenging to calculate. The section ‘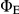 for Markovian Gaussian systems’ presents the results of various simulations, designed to illustrate the in-practice applicability of 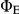 and to explore its properties. We compute 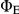 for some canonical networks, optimize connectivity under simple dynamics, and examine the numerical stability of the measure. We also compare 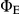 with a version of 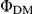 modified to apply to continuous systems, showing quantitative congruence in most cases. The section ‘Extension to multiple lags and to 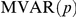 processes’ describes some additional simulation results, showing how 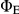 can measure integrated information over arbitrary time-steps (lags). In the section ‘Auto-regressive 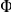 (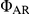)’ we describe 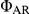 and explain its derivation in terms of relations among conditional entropy, covariance, and linear regression prediction error. We demonstrate the utility of 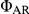 by calculating integrated information for representative systems animated by exponentially distributed (i.e., non-Gaussian) dynamics.

### Notation, conventions and preliminaries

We use bold upper-case letters to denote multivariate random variables, and corresponding bold lower-case letters to denote actualizations of random variables. Matrices are denoted by upper-case letters. The 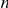-dimensional identity matrix is denoted by 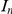 and the 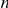-dimensional square matrix of zeros by 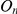. The transpose operator is denoted by ‘
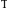
’, and the determinant by ‘det’. Our convention for logarithms is to take them to the natural base 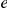, and to denote them by ‘log’.

Let 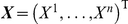 be a random variable that takes values in the space 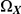. Then we denote the probability density function by 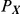, the mean by 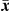 and the 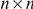 matrix of covariances, 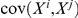, by 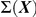. Let 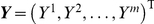 be a second random variable. Then we denote the 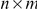 matrix of cross-covariances, 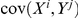, by 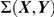. The following quantity will be useful:
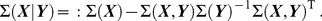
(0.1)We call this the partial covariance of 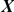 given 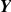, and it is well-defined when 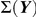 is invertible. If 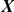 and 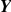 are both multivariate Gaussian variables then the partial covariance 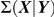 is precisely the covariance matrix of the conditional variable 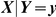, for any 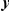:
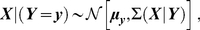
(0.2)where 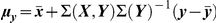.

Entropy 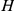 characterizes uncertainty, and is given by
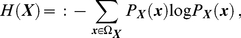
(0.3)if 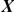 is a discrete random variable, or
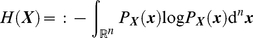
(0.4)if 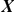 is a continuous random variable. (Note, strictly, Eq. (0.4) is the differential entropy, since entropy itself is infinite for continuous variables. However, considering continuous variables as continuous limits of discrete variable approximations, entropy differences and hence information remain well-defined in the continuous limit and may be consistently measured using Eq. (0.4) [16]. Moreover, this equation assumes that 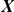 has a density with respect to the Lebesgue measure 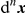; this assumption is upheld whenever we discuss continuous random variables.)

We write 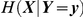 for the conditional entropy of 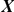 given that 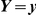, and 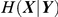 for the expected conditional entropy of 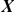 given 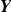, i.e.,
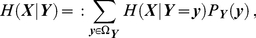
(0.5)if 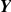 is discrete; for continuous 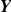 replace the summation by integration. The mutual information 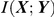 between 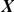 and 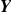 is the average information, or reduction in uncertainty (entropy), about 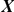, knowing the outcome of 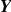:
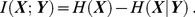
(0.6)Mutual information can also be written in the useful form
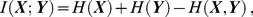
(0.7)from which it follows that mutual information is symmetric in 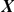 and 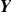 [16]. If 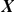 and 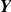 are both Gaussian,
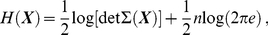
(0.8)

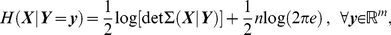
(0.9)

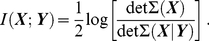
(0.10)All these quantities are straightforward to compute empirically from the empirical covariance matrices 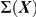 and 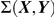, and the expression (0.1).

The Kullback-Leibler (KL) divergence 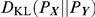 is a (non-symmetric) measure of the difference between two probability distributions 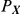 and 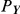 (well-defined when the variables take values in the same space, 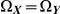). It is given by
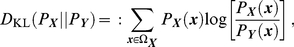
(0.11)if the variables are discrete, or
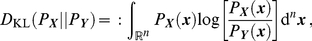
(0.12)if the variables are continuous.

We examine integrated information generated by systems of interconnected dynamical elements. We use the letter 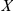 to denote such a system, and the number of elements in the system is denoted by 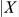. A partition 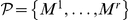 divides the elements of 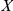 into non-overlapping, non-trivial sub-systems, 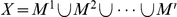. The state of 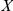 at time 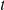 is a 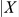-dimensional random vector denoted by 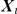, with entries corresponding to states of individual elements of 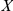. Time is discretized, so 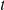 takes integer values. We denote the set of possible states of 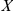 by 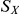, and the size of this set by 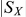. Analogous notation is used for the states of sub-systems of 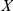.

A stationary system is one for which the probability density function for 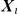 does not change with time 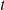. For such systems 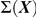 denotes the stationary covariance matrix, and 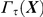 the auto-covariance matrix with time-lag 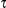:
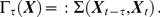
(0.13)


### The previous measure, Φ_DM_

In this section we review, following Ref. [11], the most recent version of 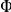 within integrated information theory, using our current notation. This measure, which we call 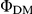 (‘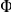-discrete/Markovian’), was defined for discrete, Markovian systems, i.e. systems with (i) a discrete set of possible states, and (ii) dynamics for which the current state depends only on the state at the previous time-step. After laying out the formal description of 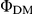, we briefly discuss these limitations, which motivate our new measures 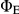 and 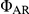.

Let 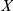 be a discrete, Markovian system. 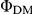 compares the information generated by the whole system to information generated by its parts, when the system transitions to a particular state 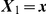 from a preceding state 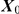 characterized by the maximum entropy distribution for the system. This is performed by use of KL divergence to compare (i) the conditional probability distribution for the preceding state of the whole given the current state; (ii) the joint distribution for the preceding states of parts given their respective current states.

The *effective information*, 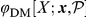, generated by 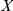 being in state 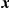, with respect to the partition 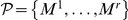, is given by
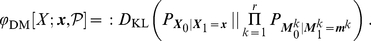
(0.14)Here 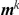 is the state of the 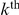 sub-system of the partition when 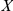 has state 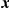.

To specify the probability distributions in (0.14), one must use Bayes' rule. For the distribution of the whole system the formula is
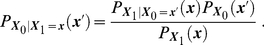
(0.15)Here 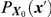 is the maximum entropy distribution, so
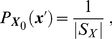
(0.16)for all possible initial states 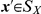. 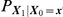 is the conditional probability density for the state at time 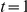 given that the state at time 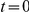 is 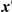. Given a generative model of the system, this distribution can be derived analytically by examining the transitions allowed by the model. In the absence of a generative model the distribution can be obtained by empirical measurement of the equivalent distribution 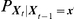. Note that in neither case is perturbation of the system required, although in the latter case the system must visit all possible states multiple times to allow reasonable estimation of 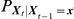. Finally, the denominator 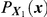 is computed from
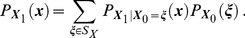
(0.17)For a part 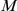 the analogous Bayes' rule formula is
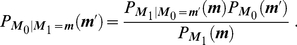
(0.18)Here 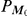 is the maximum entropy distribution on 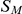. To compute the conditional probability distribution 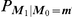 for the state at time 1 given the state at time 0 it is necessary to average over states external 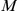. Let 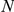 denote the complement of 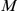 within 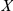, so 
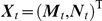
. Then we have
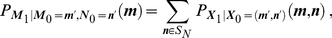
(0.19)

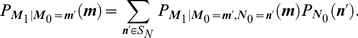
(0.20)(Note that in Ref. [11] 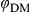 is instead computed using a perturbed version of the sub-system 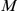, for which the joint distribution of the noise in all the afferent connections (‘wires’) to 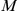 is taken to be maximum entropy. Here we instead assign the maximum entropy distribution to *states* external to the sub-system. By doing so, we eliminate the step of perturbing sub-systems, and need only perturb the whole system once, namely to impose the maximum entropy distribution as the initial state of the whole system. This choice enables simpler notation and description and does not affect the qualitative behavior of the measure [11].) Finally, 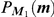 is given by
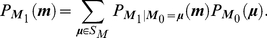
(0.21)Given the probability distributions 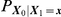 and 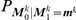, 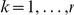, the effective information is computed using the formula (0.11) for the KL divergence.

The *integrated information* is defined as the effective information with respect to the minimum information partition (MIP). The MIP, 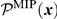, is defined as the partition that minimizes the effective information when it is normalized by
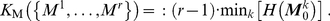
(0.22)Normalization is necessary because sub-systems that are almost as large as the whole system typically generate almost as much information as the whole system. Therefore, without normalization, most systems would have a highly imbalanced MIP, (e.g., one element versus the remainder of the system) and a trivially small value for integrated information. The normalization 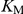 ensures that integrated information is specified using a partition defined using a weighted minimization of the effective information, with a bias towards partitions into sub-systems of roughly equal size. We will discuss the importance of normalization further in the section ‘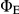 for Markovian Gaussian systems’. Thus, 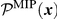 is given by
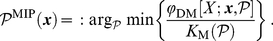
(0.23)Given the MIP, the integrated information 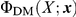 generated by the system 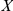 entering state 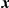 is simply the *non-normalized* effective information with respect to the MIP,
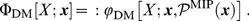
(0.24)Importantly, the value of 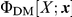 is furnished by the non-normalized effective information because it is supposed to represent a physically meaningful property of the system in the corresponding ‘integrated information theory’ [14].

For a state-independent alternative to 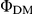, one can replace the effective information with its expectation with respect to the current state 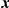, and define the *expected integrated information*, 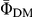, as the expected effective information across the partition that minimizes the normalized expected effective information [11]. The expected effective information, 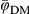, is given by [11]

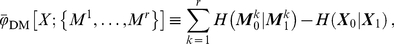
(0.25)or equivalently
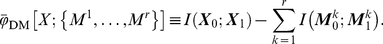
(0.26)Note that the second expression (0.26), but not the first (0.25), requires that 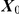 have the maximum entropy distribution [11]. To derive (0.26) from (0.25), one uses that the maximum entropy distribution is uniform, so that
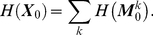
(0.27)This ensures that one can add 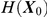 to the second term on the RHS of (0.25) and subtract 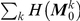 from the first term, and then use Eq. (0.6) to obtain the expression (0.26).

We emphasize that 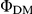 was defined only for systems that are both discrete and Markovian. The measure can not be applied to continuous systems (except those with a compact i.e. closed and bounded set of states) because there is no uniquely defined maximum entropy distribution for a continuous random variable defined on the real number line [16]. (In fact, the measure is also not applicable to discrete systems with an infinite set of states.) 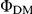 can only be applied to Markovian systems because for a non-Markovian system it is not clear how to impose the maximum entropy distribution as an initial condition, implying that the conditional probability distribution 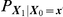 cannot be uniquely specified by any generative model. For instance three alternatives are (i) to make all past states independent and maximum entropy; (ii) to set all past states to zero except the most recent; (iii) to just set one past state to maximum entropy and obtain the distribution for other past states from the generative model. There is no immediately apparent way to choose among these alternatives. Taken together, these limitations are important because complex (e.g. neural) systems are typically non-Markovian, and neural signals are often recorded as continuous variables. In ‘Methods’ we describe an extension to 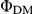 that renders it well-defined for stationary continuous, but still Markovian, systems by choosing a maximum entropy distribution based on the stationary variances of the states of individual elements. This enables us to compare 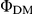 with our new measure 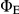 for some example cases.

### The new measure, Φ_E_

#### The general case

In this section we introduce a new measure of integrated information, 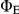, constructed analogously to 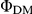, but with modifications to broaden its applicability, both in theory and in practice. 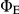 is designed for stochastic stationary systems, for which it provides a single time- and state-independent value (given a timescale of measurement, discussed below). The measure is particularly easy to apply to stationary Gaussian systems, either from time-series data or from a generative model.

The key modification is that rather than measuring information generated by transitions from a hypothetical maximum entropy past state, 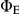 instead utilizes the actual distribution of the past state; hence the name 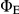, ‘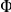-empirical’. This ensures that the measure does not suffer from the in-principle restrictions that pertain to 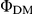, and can be applied to both discrete and continuous systems with either Markovian or non-Markovian dynamics. (More specifically, 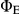 will be well-defined as long as the states 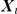 of the system are either discrete or have continuous probability densities with respect to a Lebesgue measure 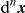.) A second difference is that, in order to be state-independent, 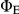 is based on the *average* information generated by the current state about the past state, as opposed to information generated by a particular current state. Finally, 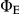 is defined so as to enable a choice of timescale (indicated by 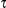) over which integrated information is measured. Thus 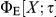 is the integrated information generated by the current state of the system about the state 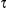 time-steps in the past.

We now define 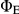 for a stochastic system with stationary dynamics. As for 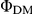, 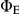 is defined via ‘effective information’. For the new measure we define the effective information generated by the current state 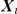 about the state 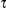 time-steps ago, with respect to bipartition 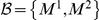, to be the mutual information generated by the whole system minus the sum of the mutual information generated by the parts within the bipartition. Thus
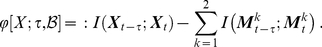
(0.28)


The integrated information 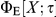 is then the non-normalized effective information with respect to the minimum information bipartition (MIB),
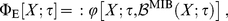
(0.29)where
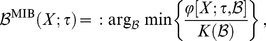
(0.30)and
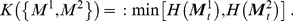
(0.31)



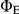
 can either be computed analytically from a generative model, or estimated numerically from time-series data. In either case, one must first obtain estimates of the probability distributions for the states 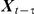 and 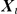, and their joint distribution 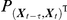, as well as the corresponding distributions for all sub-systems. Then, given these distributions, the corresponding entropies can be computed using Eq. (0.3), for a system with discrete states, or Eq. (0.4) for a system with continuous states. Having obtained these entropies, Eq. (0.7) can be used to obtain the mutual information 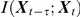 between the past and current state of the system, and likewise for all sub-systems. Given these quantities, 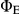 can then be obtained directly from Eqs. (0.28)–(0.31).

For numerical computation, the required probability distributions can in principle be obtained directly from data, although in practice it may be difficult to obtain sufficient data to enable accurate estimation of all the relevant entropies. As we explain in the section ‘Computing 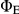 empirically under Gaussian assumptions’, this difficulty can be readily overcome if states are Gaussian distributed.

For analytic computation of 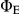 given a generative model, we note that the probability distributions for 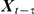 and 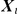 *individually* are both simply equal to the stationary distribution for the state of the system. Obtaining the joint distribution for 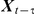 and 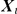 *together* will depend on the details of the generative model. Once again the situation is much easier in practice for Gaussian systems, in which case only the covariance matrix of each probability distribution is needed (see equation (0.8)). As we show in the section ‘Computing 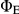 analytically for a Gaussian system’, these matrices can be derived easily from a generative model expressed as a generalized connectivity matrix, assuming Gaussian dynamics.

A few further remarks about 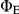 are worth making. First, that 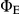 remains well-defined as a time-dependent quantity for non-stationary stochastic systems; we focus on the stationary case for simplicity, and because of our interest in empirical measurement of 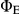 via sampling from time-series data. Second, unlike 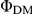, 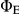 is not defined for deterministic systems. This is because it does not incorporate a perturbation through which to introduce probabilities into a deterministic system. Third, we restrict attention to bipartitions for computational efficiency. This is standard practice for computing 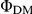 [11], [14]. Extension to general partitions is trivial, albeit computationally expensive. Finally, since mutual information is symmetric in its two arguments (0.7), effective information as given by (0.28) can alternatively be read in terms of information generated by the past state 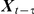 about the current state 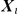.

Our definition (0.28) for the effective information, 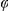, is based on the expression (0.26) for the *expected* effective information, 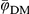 in the construction of 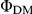. A viable alternative would be to instead use
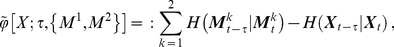
(0.32)the analogue of (0.25). This quantity has previously been defined in Ref. [22] as ‘stochastic interaction. It is the average KL divergence between (i) the past of the whole given the present of the whole, and (ii) the product of this for parts [11]. Replacing 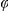 with 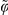 in the definition of 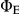 leads to a second measure 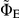. In general, 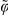 will not be exactly equal to 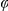. (Equality of their analogues for 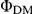 relies on the past state being maximum entropy, see section ‘The previous measure, 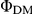
’.) However, we show in Table 1 that 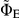 behaves very similarly to 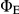 for the examples we consider in this paper. We choose to focus on 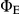 because it explicitly operationalizes the concept of ‘information generated by the whole minus the sum of information generated by the parts’ (0.28).

**Table 1:** Integrated information computed in various ways for the networks shown in Figs. 1 and 2.

Network	(i) 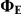	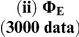	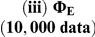	(iv) 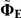	(v) 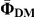	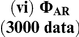
1(a)	0.0323	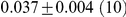	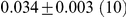	0.0323	0.0323	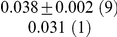
1(b)	0.0645	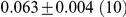	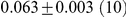	0.0645	0.0645	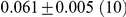
1(c)	0.1283	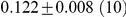	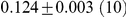	0.1387	0.1313	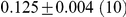
1(d)	0.0795	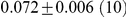	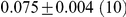	0.0894	0.0755	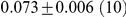
1(e)	0.1285	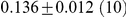	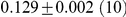	0.1376	0.1303	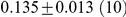
1(f)	0.1294	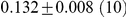	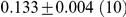	0.1383	0.1307	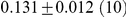
1(g)	0.1266	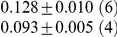	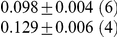	0.1362	0.1288	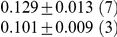
2(a)	0.2502	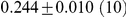	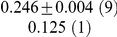	0.2652	0.1254	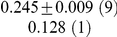
2(b)	0.2965	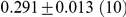	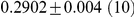	0.3012	0.2647	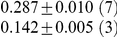

Methods of computation are (i) 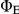 computed analytically; (ii) 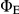 computed numerically from 10 trials of 3000 data points each; (iii) 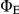 computed numerically from 10 trials of 10,000 data points each; (iv) 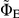 computed analytically; (v) (extended) 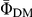 computed analytically, and (vi) 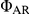 computed numerically from 10 trials of 3000 data points each, with the noise exponentially distributed. For numerical computation, means and standard deviations are given; the number of trials resulting in each value is given in parentheses. In all cases 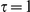.

In summary, we have defined a new measure of integrated information 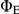 that is broadly well-defined, and which is easy to measure under Gaussian dynamics, either from time-series data or given a generative model (see below). In contrast, the previous measure 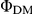 is only defined for discrete, Markovian systems. As a consequence, 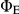 but not 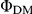 is applicable to realistic continuous non-Markovian stochastic models of neural systems.

#### Computing 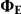 empirically under Gaussian assumptions

Under Gaussian assumptions, equation (0.10) furnishes an expression for 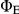 simply in terms of covariance matrices, enabling straightforward empirical computation. The effective information is given by
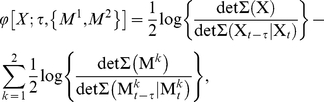
(0.33)and the normalization factor 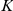 by
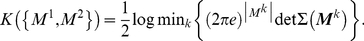
(0.34)In practice, the procedure for computing 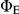 is as follows. First one obtains empirically the covariance matrices 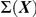, 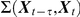 and analogues for all sub-systems. Then one uses Eq. (0.1) to obtain the partial covariance 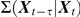 and its sub-system analogues. Given these quantities, equations (0.33) and (0.34) furnish estimates for the effective information and normalized effective information with respect to any given bipartition. These estimates allow identification of the MIB and 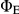, via equations (0.29) and (0.30).

#### Computing 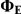 analytically for a Gaussian system

In this section we describe analytical computation of 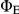 for Gaussian systems, assuming that the generative model is known. We first recognize that a generative model for a Gaussian stationary system is always equivalent to an 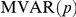 (multivariate auto-regressive) process [18]

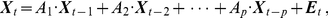
(0.35)where the 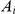, 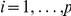, can be understood as generalized connectivity matrices acting at different time-lags, and 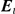 is a stationary multivariate Gaussian ‘white noise’ source with zero mean and vanishing auto-covariance function, 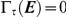, 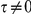. (Technically, there also exists the case 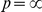, but we do not consider this here, because in practical application there will always be an optimal range of finite 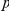 for model fitting.) Below, we show how to calculate 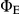 for an MVAR(1) system at timescale 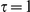. Extension to the general 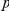, general 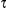 case is given in the ‘Methods’ section. Consider the generative model
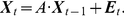
(0.36)Taking the covariance of both sides of (0.36) gives
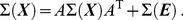
(0.37)Noticing that this equation is the discrete-time Lyapunov equation, 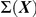 can be computed numerically, given 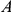, for example, in Matlab via use of the ‘dlyap’ command. To compute the partial covariance 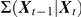 we need the single time-step auto-covariance matrix
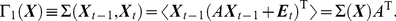
(0.38)We can then use equation (0.1) to obtain the partial covariance as
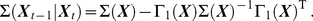
(0.39)Having values for 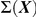 and 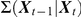 allows calculation of the first term in the RHS of (0.33). Calculation of the second term, and of the normalization factor, requires consideration of sub-systems. For a sub-system 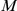, we consider the bipartition 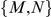, and the block decomposition of vectors and matrices according to 
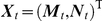
. The matrices 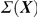 and 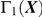 can then be written in the form
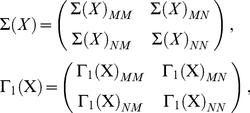
(0.40)and we can use that
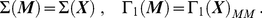
(0.41)Then, again from (0.1), the partial covariance is given by
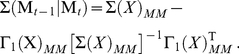
(0.42)Equations (0.37)–(0.42) together furnish the covariance matrices needed to compute the effective information and normalized effective information from the formulae (0.33) and (0.34) valid for Gaussian systems. Finally, the MIB and 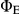 are obtained from Eqs. (0.29) and (0.30).

### Φ_E_ for Markovian Gaussian systems

#### Canonical examples

**Figure 1:**
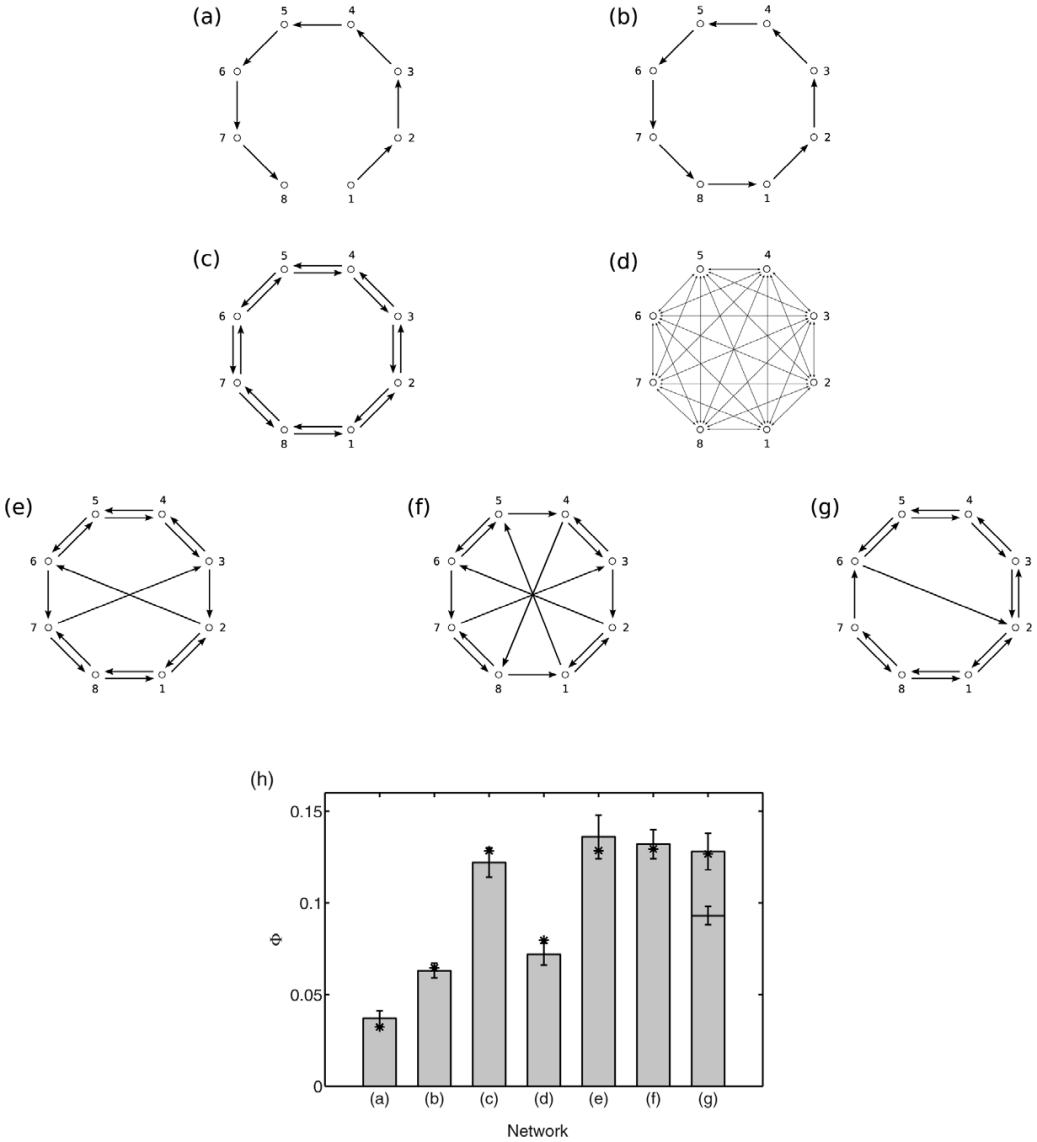
Integrated information in Markovian Gaussian systems. (a)–(g) Connectivity diagrams for seven systems as specified by the corresponding connectivity matrices 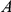. Arrow widths reflect connection strengths: for (a)–(c) and (e)–(g), all connection strengths are 0.25; for system (d) each connection strength is 1/14, thus the total afferent connection to each element is 0.5. (h) Integrated information, as measured by 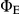 (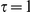) for each of the systems (a)–(g), via simulated data (bars) and analytically via the generative model (asterisks). For simulated data, 10 trials were performed, with each trial generating 3000 data points. Bars show mean values; error bars show plus/minus one standard deviation. For system 1(g), sizes of sub-systems in the MIB varied across trials, falling into two distinct groups which are shown separately (the top bar reflects a group of 6 trials; the bottom bar, 4 trials).

We present results from computing 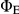, for timescale 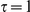, for some example Markovian Gaussian systems. Results are given for analytical computation given the generative model, and for numerical computation given simulated time-series data. The example systems are characterized by the MVAR(1) dynamics
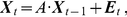
(0.43)where 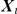 contains 8 variables, 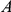 is the connectivity matrix, and each component of 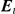 is an independent Gaussian random variable of mean 0 and variance 1. We considered seven systems, with connectivity as shown in Fig. 1(a)–(g); we refer to these systems ‘1(a)’, ‘1(b)’, and so on. The corresponding values of 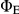 are given in Fig. 1(h) and Table 1. For analytic computation, we performed the procedure described in the section ‘Computing 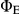 analytically for a Gaussian system’. For simulated measurements, we first obtained time-series data from equation (0.43), and then computed 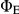 using the recipe described in the section ‘Computing 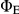 empirically under Gaussian assumptions’. To examine numerical stability of simulation measurements, we performed 10 trials for each network with 3000 post-equilibrium data points and a separate set of 10 trials with 10,000 post-equilibrium data points.

For all systems, except 1(g) (which we discuss below), the analytically derived (true) value of 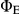 lay within 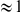 standard deviation of the mean value obtained via the simulations, both for 3000 and 10,000 data points (see Fig. 1(h) and Table 1). This correspondence confirms the consistency of the numerical and analytical approaches described above.

The values of integrated information mostly correspond with expectations. For example, a ring of reciprocal connections (1(c)) integrates approximately twice as much information as a ring of unidirectional connections (1(b)), which itself integrates approximately twice as much information as a (non-closed) chain of unidirectional connections (1(a)). Also as expected, the homogenous system 1(d) has a low 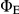 value. Perhaps in contrast to expectations, adding sparse long-range ‘short-cut’ connections to a reciprocal ring (1(e)–1(g)), in the style of a so-called ‘small world’ network [23], [24], [25], does not increase 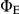 (compare with network 1(c)).

For values of 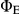 to be meaningful it is essential that they are stable with respect to numerical computation. To assess numerical stability, we calculated the coefficient of variation (the standard deviation divided by mean) across each set of 10 trials. For all networks other than 1(g), and for trial sets of both 3,000 and 10,000 data points, the coefficient of variation was less than 0.11, confirming that empirical calculation of 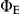 from time-series data is stable for these networks.

Network 1(g) exhibited instability when measuring 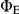 from simulation. As shown in Fig. 1(h), the corresponding values of 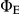 fell close to one of two values, one of which was the true (analytically derived) value. For simulations of 3,000 data points 6/10 trials produced 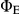 estimates close to the true value; for 10,000 data points 4/10 trials provided such estimates. This instability arises from the use of *normalized* effective information (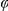) in identifying the MIB, but *non-normalized* 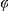 in specifying the corresponding value of 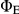. Given finite data, estimates of 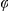 cannot be guaranteed to be accurate. As a result, inter-trial variation in measuring 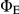 from data can arise when (i) there are two (or more) partitions with similar values of normalized 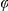 close to the true minimum (used to identify the MIB), and (ii) these partitions have substantially different values for non-normalized 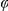. The latter condition will typically hold when partitions with similar normalized 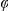 have significantly different sub-system sizes (see the section ‘The previous measure, 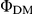
’). Network 1(g) illustrates this difficulty. For this network, the true MIB is the bipartition 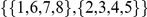, for which the normalized 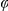 is 0.0213. However, there is an uneven bipartition, 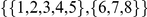 for which the normalized 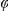 is 0.0218, i.e., very similar to the value of 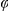 for the true MIB. However, the non-normalized 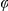 for the MIB (i.e. 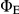) is 0.1266, whereas the value for the uneven bipartition is 0.0966. Fig. 1(h) and Table 1 show that empirical measurements of 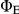 cluster around these two values.

One may consider that this problem of instability could be avoided by using non-normalized 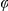 to identify the MIB. However, as discussed in the section ‘The previous measure, 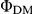
’, in this case 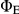 would always be trivially small because, for any non-trivial system 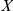, a bipartition of the form 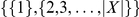 would generate almost as much information as the whole system. A second solution would be to specify 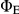 in terms of normalized 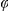. However, in this case the meaning of 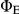 would be substantially altered inasmuch as it could no longer be considered a measure of the quantity of information generated (or integrated) by a system.

#### Optimization of networks for generating high 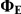


To examine whether network structures other than reciprocally connected rings could generate high levels of 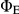, we performed numerical optimizations using a genetic algorithm (GA). Specifically, we used 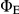 (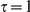) as an objective function for evolving populations of networks with dynamics governed by MVAR(1) processes (see Eq. (0.43)). We performed two sets of optimizations under different constraints on the connectivity matrix 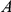. In the first set, all connection strengths were fixed (‘fixed’ condition; two afferents per element each with strength 0.25). In the second set, connection strengths were allowed to vary (‘vary’ condition; total afferent to each element equal to 0.5, all afferents to a given element equal and positive). Each condition consisted of 20 separate GAs, each with 30 randomly initialized networks in the population; (in the ‘vary’ condition networks were initialized with elements having on average 2 afferent connections). Each GA ran for 200 generations, allowing fitness to asymptote. Within each generation, the fitness of each network was determined by analytical computation of 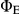; networks were then ranked by fitness and a new population was formed by rank-based selection and mutation. In the ‘fixed’ condition, each network was mutated by rearranging 2 connections; in the ‘vary’ condition each network was mutated by (with equal probability) adding, removing, or swapping 2 connections, followed by renormalization of total afference to each element to 0.5.

**Figure 2:**
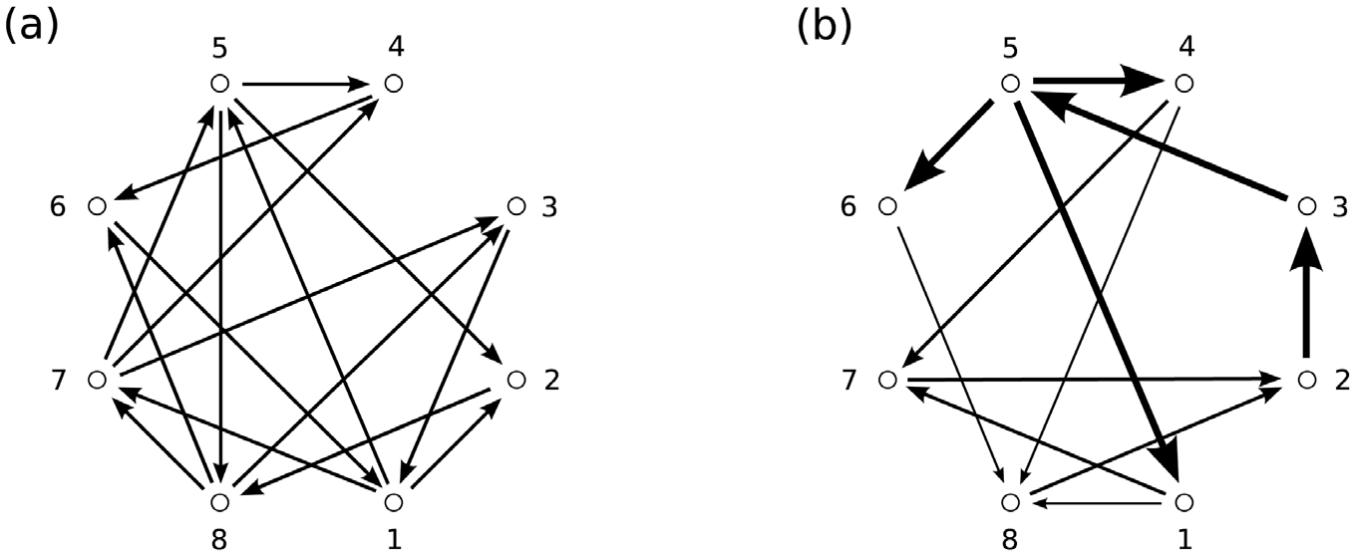
Networks optimized for high integrated information. (a) Optimal network for 2 afferents of 0.25 to each node. This has 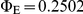. (b) Optimal network for total afferent of 0.5 to each node, and all connections to a given node equal. This has 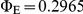.

The results of the optimizations are shown in Fig. 2 and Table 1. Network 2(a) is the fittest (highest 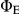) across all 20 GAs in the ‘fixed’ condition; this network topology was discovered by 6 out of the 20 GAs in this condition. The network has 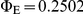, approximately twice the value of the reciprocal ring networks shown in Fig. 1. Network 2(b) is the fittest across all 20 GAs in the ‘vary’ condition, exhibiting 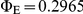, i.e., substantially higher again. This particular topology was discovered by only 2/20 GAs, perhaps due to the larger search-space in this condition. It is noteworthy that both of these ‘fittest’ networks show highly heterogeneous connectivity patterns, consistent with the intuition that integrated information is characterized by the coexistence of differentiated and integrated dynamics.

**Figure 3:**
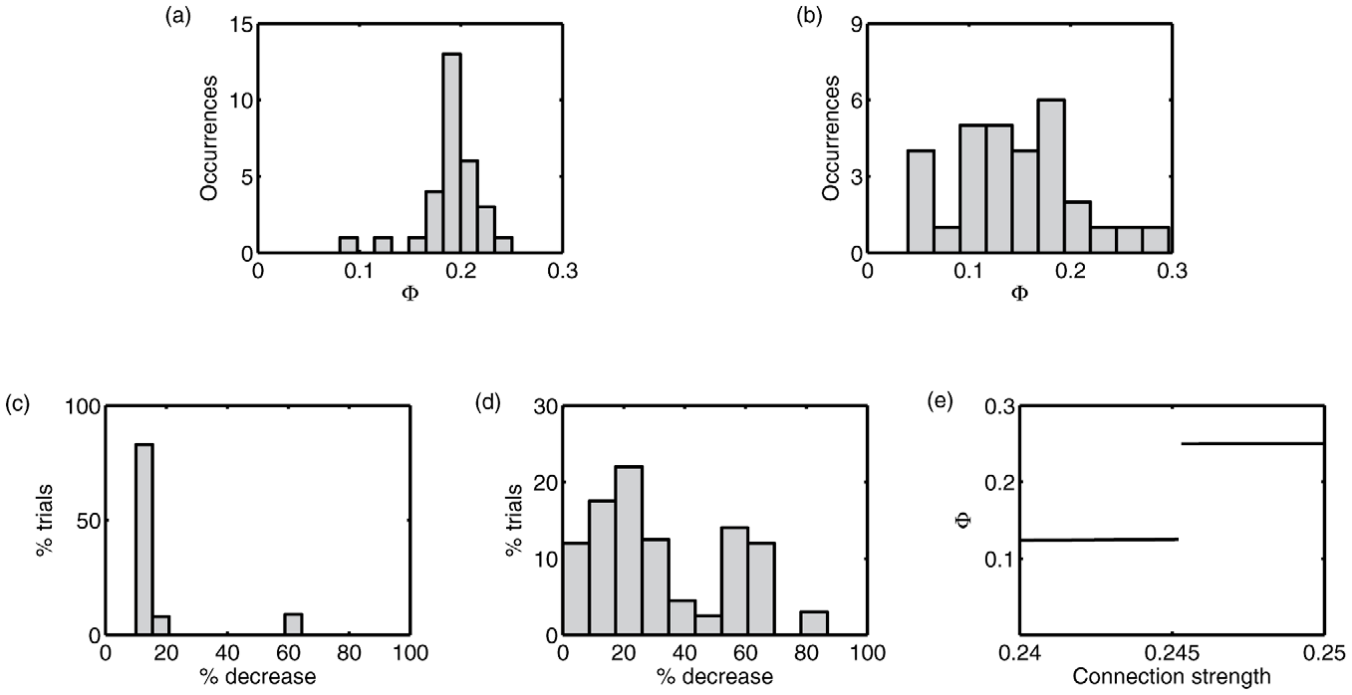
Examination of the 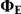 landscape with respect to network connectivity. (a) Histogram of 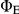 for the 30 networks in the final population of a GA that yielded optimal network 2(a). (b) Histogram of 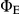 for the 30 networks in the final population of a GA that yielded optimal network 2(b). (c) Histogram of percentage decrease in 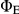 following single mutations of network 2(a) (200 evaluations). (d) Histogram of percentage decrease in 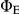 following single mutations of network 2(b) (200 evaluations). (e) Discontinuity in 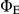 as connection strength from element 6 to element 1 continuously changes (network 2(a); (all other connections fixed at 0.25)).

The observation that the fittest network found in each condition was only reached by a minority of GAs suggests that the 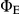 landscape across MVAR(1) systems has local maxima and may exhibit ruggedness and discontinuities. To characterize this landscape, we first plotted the distribution of fitness values across all networks in the final populations from GAs that yielded the (fittest) networks 2(a) and 2(b). Figs. 3(a,b) show that in both cases the modal value of 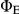 was substantially less than the maximum value, indicating a lack of convergence suggestive of local maxima and/or ruggedness [26]. We next examined the sensitivity of 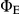 to single mutations. Figs. 3(c) and 3(d) show the percentage decrease in 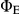 following 200 separate mutations of networks 2(a) and 2(b) respectively (the corresponding mutation type was used in each case, i.e., ‘fixed’ for 2(a) and ‘vary’ for 2(b)). For network 2(a), post-mutation fitness decreases cluster in the range 10–20%, with a few instances of 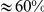. For network 2(b), more than 20% of mutations resulted in a fitness decrease of 50% or more. Together, these observations show that the value of 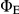 generated by a network is highly sensitive to small changes in topology and connection strength, further pointing to the ruggedness of the 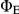 landscape.

The instability arising from using normalized effective information to find the MIB, (see ‘Canonical examples’), suggests that there may be discontinuities, as well as ruggedness, in the 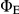 landscape. We were able to confirm the existence of such discontinuities by incrementally perturbing a specific connection in the example network 2(a). The MIB for this network is the bipartition 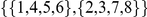, for which the normalized effective information is 0.0421. However, there is an uneven bipartition, 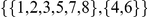 with the very similar normalized effective information of 0.0424. We incrementally weakened the connection between the two sub-systems in this uneven bipartition, finding that there is a discontinuous change in 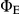 at the point at which the uneven bipartition becomes the MIB (see Fig. 3(e)).

#### Comparison with 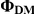, 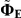, and full table of MVAR(1) results

It is instructive to compare results obtained using 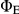 with those obtained from the version of 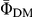 extended to apply to stationary continuous (but still Markovian) systems (see sections ‘The previous measure, 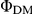
’ and ‘Methods’). Table 1 shows (extended) 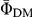 values for the various networks discussed above, as well as the corresponding 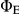 values. For networks 1(a) and 1(b) the two measures are exactly equivalent, which is explained by the stationary and maximum entropy distributions coinciding. For the remaining networks, (except network 2(a), discussed below), the two measures remain very similar, confirming 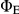 as a valid and useful measure of integrated information.

The network 2(a) has a value for 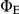 that is approximately double that of the corresponding 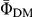. This discrepancy can also be attributed to the instability arising from normalization. Specifically, the difference between the stationary and maximum entropy distributions in this case is sufficient to lead to two different MIBs, with constituent sub-systems of different sizes. In fact, use of 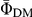 leads to the MIB 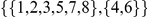 of the *perturbed* version of this network discussed in ‘Optimization of networks for generating high 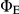
’.

We also compared results obtained using 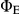 with those obtained using 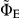, the measure constructed using the alternative expression (0.32) for the effective information (Table 1). We found that the two measures behave in qualitatively the same way across all examples.

### Extension to multiple lags and to 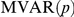 processes

**Figure 4:**
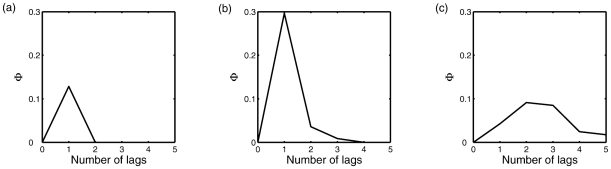
Integrated information, 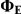 measured for states multiple time-steps in the past, i.e. for varying 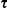. (a) Network 1(c). (b) Network 2(b). (c) Example MVAR(3) process, see Eq. (0.44).

The analyses in the previous section were concerned with integrated information measured across a single time-step for MVAR(1) processes. However, 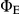 is well-defined for general 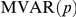 processes and can measure integrated information over any number of time-steps (lags). Here we illustrate this property using three simple examples in which 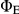 was computed analytically, via the method outlined in ‘Computing 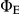 analytically for a Gaussian system’ and ‘Methods’. Fig. 4(a) shows 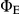 measured for various values of 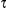, (where 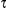 specifies the lag), for the network 1(c). Fig. 4(b) shows the same analysis conducted for network 2(b). Note that both of these networks are animated by MVAR(1) processes, which explains why 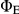 peaks at 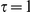 in both cases, (in other words, for these networks, most of the integrated information generated about past states by the current state is generated about the most recent past state (i.e. 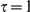)).

Fig. 4(c) shows 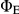 as a function of 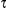 for the MVAR(3) process
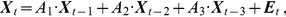
(0.44)where 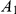, 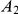 and 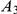 are respectively the connectivity matrices of networks 1(c), 2(b) and 2(a), each divided by 2. Note that this generalized connectivity matrix was chosen purely to provide an example of an MVAR(3) process. For this system, 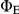 peaks at 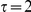, indicating that most information is integrated about the state two time-steps previous to the current state. These examples verify that 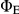 can be applied at arbitrary lags to 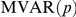 processes, and that it does detect integrated information at time-scales corresponding to a system's underlying generative mechanism.

### Auto-regressive Φ (Φ_AR_)

We have presented a measure of integrated information, 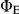, that is practical to measure from time-series data under Gaussian assumptions. However, in the case of stationary, non-Gaussian distributed time-series, 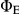 can no longer be obtained directly from empirical covariance matrices, and the required entropies must be obtained via estimation of the corresponding probability distributions. For non-trivial systems accurate entropy estimation may typically require the collection of more data than is practical.

We now describe how, even for the non-Gaussian case, the recipe used to calculate 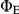 under Gaussian assumptions can nonetheless lead to a meaningful quantity reflecting integrated information. We call this quantity 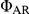 (‘auto-regressive 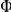
’). By construction, 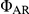 is equivalent to 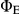 for Gaussian systems, however, for non-Gaussian systems it may differ. In all cases, because it is based on empirical covariance matrices, it remains easy to measure in practice. The motivation for considering 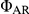 as a useful measure of integrated information rests on relations between conditional entropy, partial covariance and linear regression prediction error, explained below [17].

First we rehearse the concept of linear regression. Let 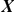 and 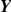 be two multivariate random variables. Then the linear regression of 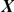 on 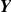 is the expression
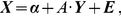
(0.45)where 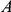 is termed the regression matrix, 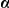 is a vector of constants, and 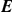 is the prediction error (or ‘residual’) [27], [28], [29], [17]. The residual is a random vector uncorrelated with 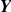. This representation is unique given the distributions of 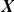 and 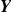, with 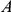 and 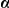 given by
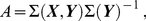
(0.46)

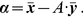
(0.47)The residual has zero mean and, importantly, its covariance matrix is precisely the partial covariance of 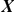 given 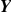 [17], thus
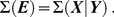
(0.48)Note that this identity holds for any 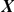 and 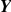, Gaussian or otherwise. For the case that 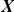 and 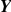 are Gaussian, we can use Eq. (0.9) to obtain, for all 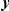,
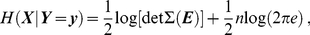
(0.49)where 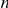 is the dimension of 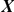. This relation between conditional entropy and linear regression prediction error implies that, for Gaussian systems, 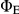 can be re-expressed in terms of linear regression prediction errors. Thus, the formula (0.33) for effective information can be re-written as
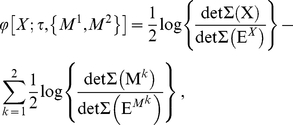
(0.50)where 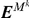, 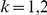, and 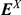 are the residuals in the regressions
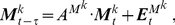
(0.51)

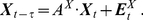
(0.52)For a non-Gaussian system, although Eq. (0.50) does not hold, its RHS nonetheless constitutes a quantity that is easy to measure empirically. This quantity forms the basis of the alternative measure 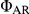, which we now define. Let 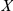 be a stationary, not necessarily Gaussian, system, and let 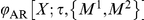 be the RHS of Eq. (0.50), i.e.
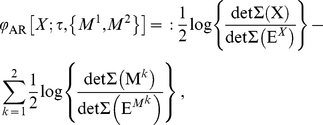
(0.53)where 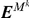, 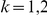, and 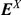 are the residuals in the regressions (0.51) and (0.52). Then 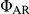 is simply 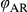 for the bipartition that minimizes 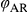 divided by the normalization factor
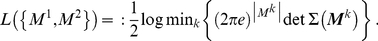
(0.54)Thus,
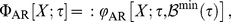
(0.55)

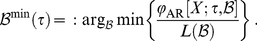
(0.56)For Gaussian systems, 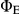 and 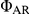 are exactly equal. For non-Gaussian systems the two measures differ, because the relation (0.49) between conditional entropy and linear regression prediction error no longer holds. However the equivalence (0.48) between partial covariance and prediction error does still hold. Hence, for *any* stationary system, the recipe for computing 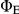 under Gaussian assumptions (as laid out in ‘Computing 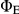 empirically under Gaussian assumptions’) yields precisely 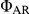. Notably, this recipe implies that it is not necessary to explicitly carry out the linear regressions; rather, the equivalence (0.48) shows that 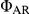 can be calculated using empirical covariance matrices.


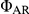
 is meaningful as a measure of integrated information because of its formulation in terms of linear regression prediction error. 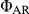 compares the whole system to the sum of its parts in terms of the log-ratio of the variance of the past state to the variance of the residual of a linear regression of the past on the present. In other words, 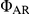 can be understood as a measure of the extent to which the *present* global state of the system predicts the *past* global state of the system, as compared to predictions based on the most informative decomposition of the system into its component parts. When Gaussian conditions are satisfied, the interpretation of 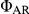 in terms of (backwards) prediction becomes exactly equivalent to the interpretation of 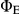 in terms of Shannon information. Note that in fact, by the symmetry of mutual information (0.7), (0.28), 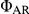 could also be expressed in terms of entirely analogous linear regressions in which the *present* is used to predict the *future*. Understood this way, 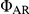 provides an interesting complement to complexity measures based on Granger causality, such as *causal density* [5], which are also based on linear regression models [30], [5], [18] (see ‘Comparison with causal density and neural complexity’).

**Figure 5:**
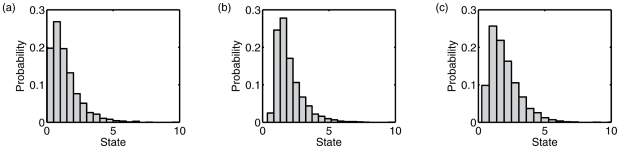
Stationary distributions for elements in networks animated with exponentially distributed noise. Each panel shows an empirical probability distribution as a histogram taken from 3000 data points from element 1 in (a) network 1(b), (b) network 1(d), and (c) network 2(b).

To demonstrate the use of 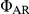 as distinct from 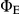, we re-animated the networks 1(a)–1(g), 2(a) and 2(b) with non-Gaussian dynamics. Specifically, we replaced the Gaussian noise sources 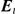 in Eq. (0.43) with independent random variables drawn from exponential distributions with mean (and variance) 1. This selection was motivated by the observation that aggregate assemblies of Poissonian spiking neurons typically follow an exponential distribution [31]. Fig. 5 shows representative examples of single-element empirical stationary distributions resulting from this modified dynamics; all show a large deviation from the Gaussian. For each network we computed 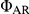 empirically from 10 trials of 3000 data points each. The results, shown in Table 1, suggest that in each case 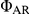 for the non-Gaussian dynamics is approximately equal to 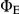 (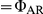) for the Gaussian dynamics. This finding provides support for 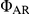 as a useful alternative to 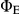, applicable to non-Gaussian dynamics.

## Discussion

In this paper we have presented two new measures of integrated information, 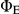 and 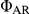. As with a previous measure, 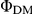, our measures quantify the information generated by a system over and above that which can be accounted for by its parts acting independently [11]. However, whereas 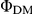 is defined only for discrete Markovian systems, and is therefore difficult to measure in practice, our quantities are well defined much more generally, and are easily applicable to stationary time-series data. Our key innovations are (i) to treat information in terms of reduction in uncertainty from the empirical as opposed to the maximum entropy distribution (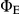), and (ii) to interpret integrated information in terms of predictive ability of the present of a system with respect to its past (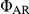). Simulations showed that our measures conform to intuitions regarding conjoined dynamical integration and segregation; where comparisons could be made, in most cases our measures quantitatively aligned with 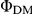. By showing how to measure integrated information from time-series data and for non-trivial non-Markovian systems, our results provide new opportunities for examining the role of integrated information in complex biological systems of all kinds, and carry implications for integrated information theories of consciousness. In the following discussion, we use the symbol 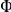 to refer to integrated information independently of its method of measurement.

### Empirical and maximum entropy distributions

As mentioned, many of the restrictions in applicability of 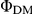 arise from the use of the maximum entropy distribution to measure information. The maximum entropy distribution is maximally agnostic with respect to the behavior of a system, and represents, in some sense, its potential, or ‘capacity’ (see ‘Integrated information as a measure of consciousness’ and ‘Comparison with causal density and neural complexity’). However, since the maximum entropy distribution typically does not arise spontaneously, it must be introduced as the distribution of a hypothetical initial state [11]. To compute 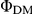 one therefore has to characterize evolution from all possible initial states of the system. However, for most practical purposes, especially in biology, it is only possible to experimentally examine systems in the context of their ongoing evolution as a sequence of states. Unless the system is Markovian, evolution from a state with history is not the same as evolution from a hypothetical initial state, implying that 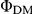 cannot be applied to non-Markovian systems (with the exception of idealized simulated systems for which a separate generative model can be written down for evolution from the initial state). Equally important, but easier to appreciate, is that it is not possible to apply 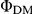 to continuous systems (except those with a compact, i.e. closed and bounded, set of states) because there is no uniquely defined maximum entropy distribution for a continuous random variable defined on the real number line [16].

Our new measure 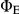 eliminates the need to consider the maximum entropy distribution by being based instead on the information generated by the current state of the system about the *actual* state of the system some number of time-steps in the past. This approach lifts the conditions that the system be discrete and Markovian. (Note however that 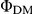 but not 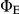 is applicable to deterministic systems, by virtue of introducing probabilities via the maximum entropy initial state.)

In principle, use of the empirical distribution de-emphasizes the notion of ‘capacity’ because the generation of information is measured with respect to what the system *has done* rather than what it *could do*. However, over large samples and for ergodic systems, this distinction becomes increasingly blurred. In practice, computing 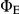 via sampling from time-series requires the data to be stationary. We recognize that not all complex biological systems generate stationary dynamics (see, e.g., Ref. [32]). However, stationarity is a common pre-requisite for statistical analysis of time-series data [33], and neural data can often be brought into this form, for example by detrending, taking first-differences and/or binning observations into short time windows [34]. Furthermore, neural dynamics are often characterized as a series of ‘metastable’ states [35], [36], [37], each of which may be locally stationary. Stationarity can also depend on the spatiotemporal granularity of observation. Dynamics that appear non-stationary at one time scale may exhibit stationarity when sampled over different time scales, underlining the principle that data acquisition should be guided by the constraints of subsequent analysis methods.

Use of the empirical, rather than maximum entropy distribution also changes the means by which 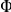 is computed. To compute 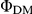, one requires the conditional probability distributions for the past state given the present state, but with an *a priori* maximum entropy distribution on the past state. Because of the maximum entropy condition (which represents ‘perturbation’ of the system), these distributions cannot be obtained empirically, but they can be obtained by applying Bayes' rule given a forward dynamical model estimated from the data (i.e. conditional probability distributions for the present state, given the past state). By contrast, computation of 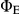 does not require Bayes' rule because, in the absence of (maximum entropy) perturbation, one can obtain the full joint distribution for the past and present directly from the data.

### Practical applicability and Gaussian dynamics


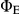
 is particularly easy to apply to data under Gaussian assumptions. This is because the relevant entropies can be estimated directly from empirical covariance matrices. It is also possible to compute 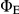 analytically from a generative model for a Gaussian system, (i.e., to any desired level of accuracy, without explicitly simulating or observing its dynamics); in that case, one obtains the necessary covariance matrices analytically. This means that 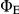 can be evaluated in practice for a broad range of biological systems.

While Gaussian dynamics are common in biology (and the assumption of Gaussianity even more so), many systems depart from this assumption. For example, the spiking activity of populations of neurons typically exhibit exponentially distributed dynamics. For the non-Gaussian case, one can still in principle calculate 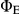 by obtaining the necessary entropies directly from data. However, in practice, accurately obtaining all of the underlying probability distributions may typically require the collection of more data than is practical. To overcome this, we introduced the second measure 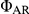. This is constructed analogously to 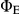, but with information replaced by the reduction in the generalized covariance of the past state under prediction via linear regression on the current state. 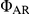 is interpreted as measuring how well the present state of a system predicts some previous state, but only to the extent that predictions based on the whole outstrip predictions based on parts independently. 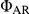 and 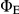 are equivalent for Gaussian systems, but otherwise differ; (recall however that 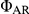 can be obtained for any system by using the recipe for computing 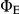 for a Gaussian system). In our examples, 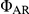 was in fact insensitive to a change from Gaussian noise to exponentially distributed noise, supporting its use as an alternative to 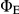.

### Normalization and instability

All versions of 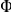 require a normalization step. Specifically, 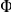 is determined by the *non-normalized* effective information (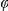) across a minimum information bipartition (MIB) which is specified as the bipartition which minimizes the *normalized* 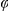 (the informational ‘weakest link’). Normalization enforces a bias towards bipartitions consisting of sub-systems of roughly equal size. Without normalization, MIBs would typically divide systems into single elements versus the remainder of the system, leading to trivially small values of 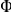. On the other hand, it remains important to determine the value of 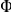 using the non-normalized 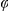 in order to allow 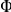 to be interpreted as a quantity of information.

The use of normalization, as just described, leads to instabilities. Our simulations have shown that 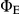 can be (i) discontinuous under a continuous perturbation of dynamics, and (ii) highly sensitive to the accuracy of entropy estimation from finite data. In our examples, these instabilities arose precisely when there were multiple partitions with similar values of normalized 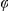 close to the true minimum *and* these partitions had substantially different values of non-normalized 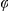. This instability does not arise for all systems, and indeed for most of our examples 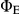 is numerically stable. Nonetheless, the embedding of normalization within the definition of 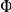 challenges ascription of physical meaning to any measured value of 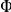. This is because the value of 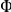 is in all cases dependent to some arbitrary degree on the normalization process involved in determining the MIB.

### Integrated information as a measure of consciousness

Previous measures of integrated information (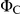 and 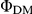) were formulated in the context of a theory of consciousness, the ‘integrated information theory of consciousness’ (IITC). According to the IITC, consciousness *is* integrated information, and has the status of a fundamental property of the universe, equivalent to mass, charge, and the like [14]. On this theory a low value of integrated information would correspond to a low conscious ‘level’ (e.g., coma, general anesthesia, deep dreamless sleep) and a high value to normal conscious wakefulness. If one subscribes to the theory using 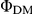, then one must interpret consciousness (integrated information) as a function of state transitions [11]; accordingly, one cannot ask about the conscious level of a system *per se*. By contrast, if one applies 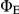 or 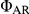 to a stationary system then they are state-independent and so, subscribing to the IITC with these measures involves viewing integrated information as a property of the system's dynamics. This in turn would imply that (i) conscious level is constant during each stationary epoch in brain activity, and (ii) conscious level changes when functional connectivity changes, modifying the stationary statistics. This view recalls William James' notion of consciousness as a process [19] and is consistent with a large amount of empirical evidence showing correlations between conscious level and plausibly stationary epochs of brain activity. For example, normal conscious wakefulness is characterized by low-amplitude high-frequency oscillations in the cortical EEG [38], whereas epileptic absence seizures are characterized instead by increased synchrony in thalamocortical systems [39]. As mentioned in the section ‘Empirical and maximum entropy distributions’, neural dynamics may be metastable [35], [36], [37], with locally stationary periods corresponding to a conscious state with a particular level and content. Our results now make it possible to measure the integrated information corresponding to these various states and to compare these values with other indices of consciousness, both subjective (e.g., verbal reports, confidence ratings, etc.) and objective (e.g., EEG synchrony, widespread brain activity, etc.) [40]. Importantly, it is now possible to quantitatively compare integrated information with other measures of neural dynamics that operationalize in different ways the notion that consciousness conjoins dynamical integration and differentiation, such as ‘causal density’ [41] and ‘neural complexity’ [8] (see ‘Comparison with causal density and neural complexity’).

An important feature of the IITC as previously expressed is that consciousness *qua* 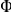 is best considered as a capacity (equivalently a potential, or disposition), and not as an ‘object’ or a process [14]. The original 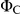 operationalized the notion of capacity by subjecting a system to all possible perturbations and examining its responses. The recent 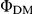 measures information as a reduction in entropy from the maximum entropy distribution, which can be taken to correspond to the capacity of a system. However, because 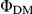 is specified by state transitions it is not a ‘pure’ measure of capacity; rather, it is a measure of capacity modulated by a system's dynamics. By measuring 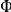 with reference to the stationary distribution, our measures depart from the notion of consciousness as a capacity. The stationary distribution characterizes the capacity of a system only to the extent that it is realized in the system's behaviour. 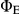 and 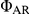 can therefore be construed as measures of a process modulated by capacity, aligning more closely with the Jamesian intuition.

The notion that 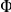 exists as a ‘fundamental property’ deserves comment. As described in the section ‘Normalization and instability’, our results challenge the ascription of physical meaning to 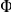, in virtue of its exquisite sensitivity to the normalization process involved in specifying the MIB: this challenge pertains equally to the notion of 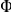 as a ‘fundamental quantity’. A further challenge to the ascription of physical meaning to 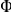 is the fact that it is not invariant under a change of coordinates, since this leads to a different set of sub-systems over which to minimize the effective information. An interesting question for future work is to examine whether, under certain conditions, the set of coordinates that maximizes 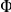 could be taken to define ‘natural’ coordinates, or macroscopic variables, for the system. In any case, it does not seem necessary to consider 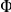 as a strict physical quantity in order to measure the integrated information corresponding to a system's state transitions or stationary dynamics, nor to relate these measurements to conscious level and content. In other words, one can depart from the IITC by interpreting 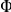 as accounting for particular aspects of consciousness without the further step of claiming identity [9].

### Integrated information in other neurocognitive processes

Although 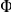 was originally developed in the context of a theory of consciousness, it is plausible that integrated information, and (more generally) conjoined functional integration and differentiation, play key roles in other cognitive and neural processes. Previous formulations (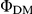, 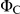) are poorly suited to investigating these roles, not only because of practical inapplicability, but also because they characterize integrated information in terms of capacity rather than process. Whereas consciousness under some theories may be considered as a capacity (see above), neurocognitive properties in general are best considered as processes. Having a measure of 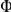 that is framed in terms of process, and that is easy to apply in practice, therefore permits the framing of testable hypotheses, and the specification of synthetic models, aimed at examining the role of integrated information in neurocognitive processes broadly construed. For example, multi-modal binding and perceptual categorization [20], and action selection (decision making) [21] plausibly involve integrated information and could be profitably analyzed using our methods. Already, related measures of dynamical complexity (neural complexity and causal density, see below) have been correlated with the ability of simulated agents to deploy flexible behavior, suggesting a role for such dynamics in sensorimotor coordination in rich environments [6], [41]. Our results now allow integrated information to be applied in similar situations, facilitating comparative analyses.

### Comparison with causal density and neural complexity


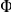
 is one among a family of recent measures that aim to characterize, in different ways, the coexistence of integration and differentiation in a system's dynamics. Two alternative measures are ‘causal density’ [41] and ‘neural complexity’ [42]. Here, we briefly summarize the similarities and differences among these measures, in order to set 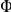 into a broader context.

Causal density, like 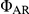 and 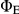 (but in contrast to 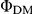 and 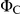), is a measure of process rather than capacity. In virtue of being based on ‘Granger causality’, it also shares with 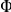 a sensitivity to causal interactions within a system. A key difference, however, is that causal density is based on *all* causal interactions, and not just those across a particular partition; thus causal density avoids the normalization problems described above (‘Normalization and instability’). Briefly, Granger causality is a statistical measure of causal influence which asserts that a variable 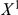 ‘Granger causes’ another variable 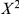 if information in the past of 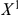 helps predict the future of 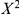, above and beyond information already in the past of 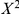 (and, optionally, in the past of a set of conditioning variables 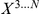) [30], [43]. Causal density is then the (weighted) fraction of causal interactions among all elements that are statistically significant. High causal density indicates that elements within a system are both globally coordinated in their activity (to be useful for predicting each others' activity) and at the same time dynamically distinct (so that different elements contribute in different ways to these predictions). Granger causality (and causal density) is typically calculated using linear auto-regressive models, which brings about an interesting comparison with 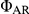. In a loose sense, integrated information, as measured by 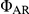 or 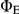, can be thought of as a variety of ‘causal density’, that quantifies the strength of the weakest bidirectional causal link between any two halves of the system. Forthcoming work will investigate further the links between 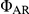 and causal density.

Neural complexity is calculated as the sum of the average mutual information across all bipartitions of a system [42]. Unlike 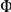 and causal density, it does not reflect causal interactions within a system, however, like causal density, it is a measure of process rather than capacity. Neural complexity is maximal in a system that is globally integrated at the level of large subsystems, while exhibiting a high degree of segregation between smaller subsystems. (Note: The original papers describing neural complexity contained an error in calculating the covariance matrix from a generative model, which has been subsequently corrected in [44]. However, it appears that this error may still affect extant calculations of 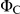.) A recent result [17] showing an equivalence between Granger causality and ‘transfer entropy’ (a time-directed version of mutual information) allows causal density to be related directly to neural complexity. Specifically, one can define a ‘bipartition causal density’ as a weighted average Granger causality (transfer entropy) across all bipartitions of a system (this definition also requires extension of Granger causality to multivariate variables) [18]. This measure furnishes a ‘time-directed’ version of neural complexity based on transfer entropy rather than mutual information.

These relations together suggest common foundations for measures of coexisting integration and differentiation. However, further work is needed to fully establish their theoretical interdependencies and their empirical convergences and divergences.

### Comparison with other measures

Characterizing complexity is a diverse field, and there are other measures that capture complex properties other than conjoined differentiation and integration. For example, ‘thermodynamic depth’ [45] can be interpreted as a measure of how hard it is to put a system together, and is based on the joint entropy of all past states, given the current state. 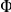 by contrast considers only one past state. An interesting further modification to 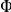 could involve information between the present and the whole past trajectory of the system. Another measure of statistical interdependence, ‘informational coherence’, considers the optimal predictive state for each time-series, and then measures mutual information between these [46]. In related work by Ay et al., the whole system is compared to the sum of individual elements [22], [47], [48], and the analysis goes beyond examination of conditional entropies to a more thorough mathematical treatment in terms of information geometry. While it is beyond the present scope to examine the formal correspondences among these measures, other related measures, and the measures described above, the growing interest in quantitative measures of complexity further emphasizes the need to formulate theoretically principled measures that are also simple to apply in practice.

### Limitations and extensions

Although our measures represent substantial improvements in practical applicability of measures of integrated information, several limitations remain. Most prominently, the normalization procedure leads to instabilities in the measurement process and undercuts ascription of physical meaning to 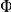. Addressing this problem stands as a key theoretical challenge. We have only considered application of our measures to stationary dynamics. Future work may extend consideration to non-stationary (but still continuous and non-Markovian) processes, potentially capturing important non-stationary aspects of neural dynamics. In addition, our measures are applicable only to stochastic systems. While extension to closed deterministic systems may be of some value, most complex biological systems have stochastic components, especially when considered in interaction with a (stochastic) environment [49], [50]. Finally, our measures share with previous measures the computational challenge posed by the combinatorial explosion in partitions of a system as the number of elements increases. Possibly, imposing priors on the search for the minimum information partition may mitigate this challenge.

We have only considered a first-order, linear approximation for computing entropies/information from data. While this is useful for drawing comparison with Granger causality and causal density, there now exist more advanced approximation techniques that could be used in future work, for example additive regression [51] or kernel regression [52]. Regarding estimation of entropy and mutual information without employing a regression model, we have only considered this via the intermediate step of density estimation. Again, future work could investigate the applicability of more advanced techniques [53], [54] that avoid this step.

As well as addressing the above challenges, future work will (i) empirically examine integrated information for time-series data acquired from neuroimaging and other biological datasets, in order to test intuitions regarding consciousness and other neurocognitive processes; (ii) investigate in models how integrated information is modulated by input and output relations of a system embedded in, and interacting with, a surrounding environment, and (iii) determine theoretically the relations between integrated information and alternative measures of dynamical complexity and metastability.

## Methods

Text S1 in ‘Supporting Information’ contains software enabling calculation of ΦE and ΦAR, as well as functions which allow regeneration of some of the simulations we describe.

### Extension and computation of Φ_DM_ for an MVAR(1) process

To extend 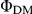 to stationary continuous Markovian systems, we have to address the problem that there is no well-defined maximum entropy distribution for such systems. We do this by replacing the ‘maximum entropy distribution’ with the distribution for which the state of each element is independent of the states of all other elements, is Gaussian distributed, and has mean and variance equal to those of its corresponding stationary distribution. Thus, we take 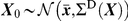, where
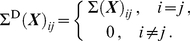
(0.57)


Having defined a distribution for the initial state 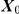, we explain how to compute the expected integrated information, 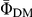, for MVAR(1) processes (0.36). The computation proceeds analytically, given the generative model, which is specified by the connectivity matrix 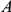 and the covariance matrix of the noise, 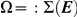. Alternatively, an estimate of 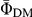 from time-series data can be obtained by using estimates of 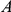 and 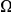. The linear-regression formulae (0.46) and (0.48) yield the estimates
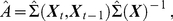
(0.58)

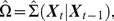
(0.59)where the symbol 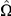 denotes empirical quantities.

Given 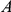 and 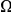, (or their estimates 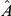 and 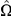), the covariance matrix 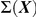 can be obtained via the discrete-time Lyapunov equation (0.37),
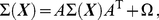
(0.60)and 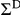 from Eq. (0.57).

To compute the conditional probability 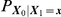 we first use the MVAR(1) dynamics (0.36) to obtain the distribution of 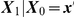 as
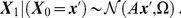
(0.61)Then we use Bayes' rule (0.15) to obtain
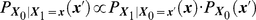
(0.62)

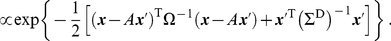
(0.63)From the term quadratic in 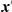 we can obtain the inverse of the covariance matrix of (the Gaussian distributed) conditional variable 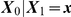 as
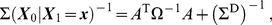
(0.64)and hence express the conditional entropy 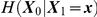 in terms of the connectivity and stationary covariance matrices:
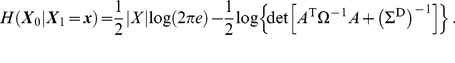
(0.65)For a given a sub-system 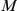, we have to consider the bipartition 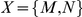, and the block decomposition of vectors and matrices according to 
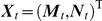
 so that
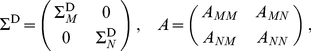
(0.66)and similarly for 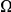 and 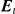. To obtain the distribution for the conditional random variable 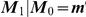, we express 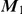 in terms of 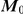 as
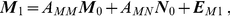
(0.67)and note that 
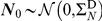
. Hence
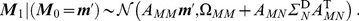
(0.68)From Bayes' rule, we can then calculate the inverse of the covariance matrix of (the Gaussian distributed) conditional variable 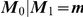 as
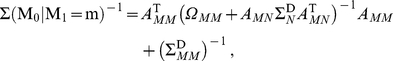
(0.69)and hence
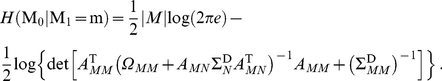
(0.70)The entropy formulae (0.65) and (0.70) furnish the sufficient quantities for computing 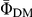 as described in the section ‘The previous measure, 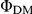
’, using the expression (0.25) for the expected effective information. For present purposes, as with 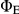, we restrict attention to bipartitions only.

### Analytical computation of Φ_E_ for a general Gaussian case

Here we show how to compute 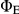 analytically, for a general stationary Gaussian system, for any timescale 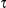. Importantly, the generative model for such a system 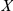 is always equivalent to an 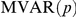 process [18]:
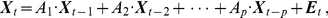
(0.71)where the 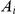, 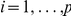, can be thought of as generalized connectivity matrices acting at different time-lags, and 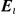 is a stationary multivariate Gaussian ‘white noise’ source with zero mean and vanishing auto-covariance function, 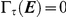, 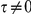. (We ignore the case 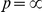 corresponding to an MA(1), i.e. moving average, process.) This system is stationary if and only if the roots of the equation
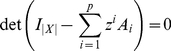
(0.72)lie outside the unit circle [33].

The method outlined in ‘Computing 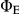 analytically for a Gaussian system’ for computing 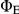 with 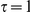 for an MVAR(1) process is easy to extend to the more general 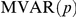, any 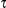, case given by equation (0.71). Suppose we wish to compute 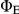 for any value of 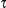 up to 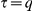, where 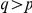. We first use the fact [33] that the 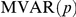 process is equivalent to the MVAR(1) process
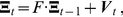
(0.73)involving the block quantities 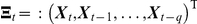, 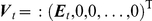 and
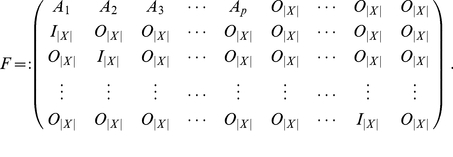
(0.74)The stationary covariance matrix 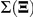 for this process can be obtained from the Lyapunov equation, by analogy with 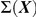 for the MVAR(1) case (0.37):
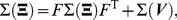
(0.75)where 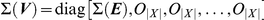. Then the stationary covariance 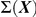 and auto-covariance 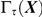 are obtained respectively as the 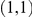 and 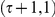 component blocks of 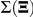. We can then proceed as for the MVAR(1), 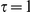 case:
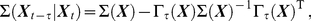
(0.76)

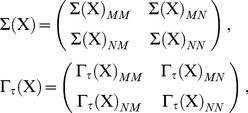
(0.77)

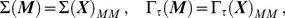
(0.78)

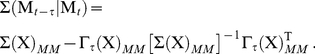
(0.79)The above expressions furnish the quantities needed to compute 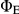 from equations (0.29), (0.30), (0.33) and (0.34).

## Supporting Information

Text S1 Toolbox for computing integrated information as Φ_E_ or Φ_AR_. ‘phiemvarp.m’ computes Φ_E_ from an MVAR(*p*) generative model. ‘ARphidata.m’ computes Φ_AR_ ( = Φ_E_ if Gaussian), from stationary time-series data. ‘statdata.m’ creates time-series data from an MVAR(*p*) generative model. ‘A2b.mat’ contains the connectivity matrix for the optimal network, Fig. 2(b). ‘timereverse.m’ is an m-file for time-reversing the data (required to run ARphidata.m).
(0.01 MB ZIP)
